# Balance Training Reduces Brain Activity during Motor Simulation of a Challenging Balance Task in Older Adults: An fMRI Study

**DOI:** 10.3389/fnbeh.2018.00010

**Published:** 2018-01-24

**Authors:** Jan Ruffieux, Audrey Mouthon, Martin Keller, Michaël Mouthon, Jean-Marie Annoni, Wolfgang Taube

**Affiliations:** ^1^Movement and Sport Sciences, Department of Medicine, University of Fribourg, Fribourg, Switzerland; ^2^Neurology Laboratory, Department of Medicine, University of Fribourg, Fribourg, Switzerland

**Keywords:** balance training, postural control, older adults, brain activity, fMRI, AO+MI

## Abstract

Aging is associated with a shift from an automatic to a more cortical postural control strategy, which goes along with deteriorations in postural stability. Although balance training has been shown to effectively counteract these behavioral deteriorations, little is known about the effect of balance training on brain activity during postural tasks in older adults. We, therefore, assessed postural stability and brain activity using fMRI during motor imagery alone (MI) and in combination with action observation (AO; i.e., AO+MI) of a challenging balance task in older adults before and after 5 weeks of balance training. Results showed a nonsignificant trend toward improvements in postural stability after balance training, accompanied by reductions in brain activity during AO+MI of the balance task in areas relevant for postural control, which have been shown to be over-activated in older adults during (simulation of) motor performance, including motor, premotor, and multisensory vestibular areas. This suggests that balance training may reverse the age-related cortical over-activations and lead to changes in the control of upright posture toward the one observed in young adults.

## Introduction

Normal aging is associated with deteriorations in postural stability (Maki and McIlroy, 1996), which eventually increase the risk for falls (Muir et al., 2010; Boisgontier et al., 2017). These behavioral impairments are accompanied by changes in the postural control strategy. Electrophysiological and imaging, as well as behavioral studies indicate a shift from an automatic, lower level control toward a more attentional, cortical control of posture (Seidler et al., 2010; Boisgontier et al., 2013; Papegaaij et al., 2014; Baudry, 2016). Older adults exhibit greater and more widespread activation of cortical areas compared to young adults when performing motor tasks (Seidler et al., 2010). Functional magnetic resonance imaging (fMRI) studies showed age-related increases in brain activity during performance of finger and coordinated hand and foot movements in prefrontal and motor areas including prefrontal cortex (PFC), premotor cortex (PMC), supplementary motor area (SMA), pre-SMA, putamen and cerebellum (Mattay et al., 2002; Heuninckx et al., 2005, 2008; Coxon et al., 2010; Goble et al., 2010; Maes et al., 2017).

Using motor imagery (MI) and action observation (AO), also standing and walking have been investigated with fMRI and similar effects of greater brain activity in older relative to young adults have been reported (Zwergal et al., 2012; Allali et al., 2014; Mouthon et al., in press). Effects were found in areas including multisensory vestibular cortices and somatosensory cortices during MI of upright standing (Zwergal et al., 2012) as well as in the SMA and frontal cortices for MI of gait (Allali et al., 2014). Most notably, using MI, AO, and the combination of the two (AO+MI) of balance tasks, we recently found over-activations in the same older adults who participated in the present study in SMA, primary motor cortex (M1), PMC, putamen and PFC (Mouthon et al., in press). In line with these imaging studies, electrophysiological measurements using transcranial magnetic stimulation (TMS) revealed that motor simulation of balance tasks was associated with greater corticospinal excitability in older compared to young adults (Mouthon et al., 2016). Importantly, age-related increases in corticospinal excitability have also been observed during actual balance tasks (Baudry et al., 2015), indicating that motor simulation is a meaningful tool to investigate postural control. These “over-activations” observed in older adults are frequently interpreted as a functional compensation for age-related declines in the structure and function of the central nervous system and thus as a positive adaptation (Seidler et al., 2010). Support for this interpretation comes from studies showing that the degree of over-activation is positively correlated with task performance in older adults (Mattay et al., 2002; Heuninckx et al., 2005, 2008; Goble et al., 2010). The increase in cortical engagement during motor performance in general and during postural control in particular, in both, motor and cognitive areas, might also explain the larger dual-task costs that have repeatedly been reported for older adults (Boisgontier et al., 2013; Ruffieux et al., 2015).

There is good evidence that balance training improves postural stability in older adults (for review see Sherrington et al., 2011). Much less, however, is known about the neural mechanisms behind these behavioral adaptations. A morphological study reported gray matter changes in the hippocampus after 6 weeks of balance training in older adults aged around 63 years (Sehm et al., 2014). Remarkably, these structural changes correlated with the improvements in balance performance. Very little is known about balance training-related changes in brain function in older adults. A recent fMRI study reported a decreased resting-state functional connectivity between the striatum and other brain areas after 6 weeks of slackline training in older adults aged around 62 years—but only in a subsample of participants who improved slackline performance (Magon et al., 2016). The authors interpreted this as an increase in efficiency of the striatal network. In an electrophysiological study, changes in spinal and corticospinal excitability were investigated in older adults using peripheral nerve stimulation and TMS, respectively, before and after 6 weeks of balance training (Penzer et al., 2015). The authors found an increase in spinal and a decrease in corticospinal excitability after training and interpreted this finding as a reversion of age-related changes in postural control toward a more automatic control.

The aim of the present study was to learn more about the effect of balance training on brain activity during performance of balance tasks in older adults. In order to investigate not only the M1 but also other cortical and subcortical brain areas, fMRI was chosen. However, since the dynamics of balance tasks do not allow fMRI measurements during the actual performance, brain activity was assessed during motor simulation of such tasks. There is clear evidence that motor simulation of a task can elicit very similar brain activity patterns than actual task execution (Jeannerod, 2001; Taube et al., 2015; Eaves et al., 2016). Two forms of motor simulation are well established: MI and AO. It has been shown that the task-related brain areas involved in these two techniques overlap to a large extent both with one another and with the areas activated during task execution (Eaves et al., 2016). While MI and AO have traditionally been used and investigated separately, there is growing evidence that combining the two techniques by instructing MI during AO activates the relevant networks even more effectively than either technique alone (Eaves et al., 2016). Accordingly, it has been shown that AO+MI of balance tasks substantially activates brain areas known to be important for postural control, including putamen, cerebellum, SMA, PMC and M1 (Taube et al., 2015; Mouthon et al., in press). In the latter study (Taube et al., 2015), MI of the same balance tasks revealed activity in similar areas except for premotor cortices and M1 while AO alone did not lead to any significant activation in the relevant areas. Using TMS during the same conditions, similar effects were found for corticospinal excitability with the largest motor evoked potentials during AO+MI, followed by MI alone and AO alone (Mouthon et al., 2015). Further evidence for the close link between motor performance and simulation comes from studies showing behavioral improvements in postural stability and walking speed after short non-physical (imagery and/or observation) training interventions, in both young and older adults (Hamel and Lajoie, 2005; Tia et al., 2010; Taube et al., 2014).

In the present study, we, therefore, investigated the effect of 5 weeks of balance training on postural stability as well as on brain activity during MI alone and during AO+MI of a challenging balance task in older adults. To the best of our knowledge, this is the first study to look at the effect of physical balance training on the neural representation of a balance task during motor simulation.

## Materials and Methods

### Participants

Thirty healthy older adults (65–80 years) with no known neurological or orthopedic disorders and normal or corrected-to-normal vision were randomly allocated to the training (mean age ± SD 70.1 ± 4.4 years, eight females, *n* = 15) or the control group (*n* = 15). Three participants of the control group did not complete the study and were excluded, leaving 12 participants in the control group (71.6 ± 5.3 years, five females). This study was carried out in accordance with the recommendations of and approved by the local ethics committee (Commission d’éthique de Recherche, Canton de Fribourg). All subjects gave written informed consent in accordance with the Declaration of Helsinki.

### Experimental Design

The participants of the training group completed a balance training of 5 weeks while the participants of the control group were requested to maintain their usual activity pattern during this time period. Before and after these 5 weeks, balance performance and brain activity patterns during MI and AO+MI of a balance task (dynamic perturbation) and a control task (unperturbed stance) were assessed in all participants. The training regimen and the measurements are described in detail below.

### Training

The balance training comprised three sessions per week for 5 weeks. It was designed as a classical balance training consisting of one-legged standing on four different unstable devices: a spinning top balance board, a tilt board, an air cushion and a foam pad. The participants performed 4–5 trials of 20 s on each leg and device. To avoid fatigue, they rested for at least 20 s between trials and 5 min between devices. The total duration of a session was about 1 h, including a warm-up and a cool-down. All sessions were supervised by an experimenter.

### Balance Performance

Balance performance was assessed during one-legged standing on the right leg in two surface conditions: on solid ground (static task) and on a free-swinging platform that is suspended on dampened springs (Posturomed 202, Haider Bioswing GmbH, Pullenreuth, Germany; dynamic task). In both tasks, the number of errors was counted. An error was defined as touching the ground with the left foot or touching the handrail of the Posturomed that was mounted to the right of the participants. The participants were instructed to minimize the number of errors and to regain the one-legged posture as quickly as possible if they conduct an error. The trials were performed barefoot with the arms akimbo while the participants were asked to fixate a mark on the wall 3 m in front of them. Two and three trials of 15 s were performed in the static and the dynamic task, respectively, and the average number of errors conducted was used for statistical analysis.

### fMRI

#### Experimental Procedure

**Figure 1:**
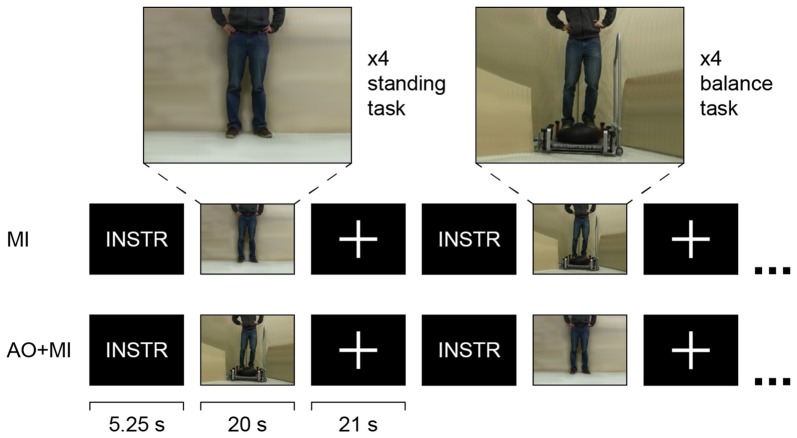
Block-design of the fMRI experiment. Participants mentally simulated a dynamic balance task (compensating a medio-lateral perturbation) and a control task (standing still on solid ground) in two conditions: motor imagery alone (MI) and while watching a video of a person performing the task (AO+MI). In each condition, four 20-s blocks of each task, separated by 21 s of rest and 5.25 s of instruction about the next block, were performed in a randomized order. In the dynamic balance task blocks, the 20 s were composed of 10 2-s sequences where each sequence represented a perturbation.

In the fMRI protocol, participants were asked to mentally simulate a balance task while lying in the scanner. The task consisted of compensating a medio-lateral perturbation while standing on an air cushion that was placed on top of the Posturomed (see Figure 1). In order to rule out any effect that was not related to the postural demand of the balance task, a second task that consisted of standing still on stable ground (standing task) was simulated and served as the control task. Thus, any brain activity that was common to the two tasks, such as activity in auditory and visual areas evoked by the picture and the sound of the videos or the sound of the scanner, could be filtered out and possible effects could be reduced to the effect of the postural challenge of the dynamic balance task. By this means, also possible attention-related effects or non-specific training effects due to aerobic exercise could be excluded. The tasks were simulated in two ways. In the MI condition, the participants were asked to imagine themselves (from a kinesthetic perspective) performing the respective task with their eyes closed. In the second condition, they performed the same MI while at the same time watching a video of a person performing the task (AO during MI, AO+MI).

In each condition (MI and AO+MI), four blocks of each postural task were performed in a fully randomized order. A block lasted 20 s, preceded by 5.25 s of visual and auditory instructions about the next condition. In the dynamic balance task blocks, the 20 s were composed of 10 2-s sequences where each sequence represented a perturbation. The start of each perturbation was signaled by a tone. This was particularly important in the MI condition where participants did not see the task (no video, eyes closed). The blocks were separated by 21-s rest periods during which a white cross on a black background was displayed on the screen in the AO+MI condition. Before each condition and each block, the participants were provided with written and verbal information about which type of simulation and task, respectively, they had to perform next (see Figure 1). The MI and AO+MI conditions were tested in separate scan runs of which the order was randomized between participants but the same for the pre- and the post-measurement in all participants.

The videos for the AO+MI condition were displayed on a 32″ MRI compatible monitor (NordicNeuroLab AS, Bergen, Norway) with E-Prime 2.0 software (Psychology Software Tools Inc., Sharpsburg, PA, USA). The videos were presented at a visual angle of 16° in the vertical and 9° in the horizontal plane. The monitor was placed outside the scanner (at the height of the participants’ feet) and participants looked at it via a mirror system. The sound was delivered by an MRI compatible audio system (Starter F Mk II package, MR Confon GmbH, Magdeburg, Germany).

The participants were carefully introduced to the tasks and familiarized with the videos before they were placed in the scanner. It was emphasized that it was crucial that they performed all the tasks only mentally, without any actual movements. The participants’ ability to imagine movements was tested by a standardized questionnaire (short version of the Kinesthetic and Visual Imagery Questionnaire, KVIQ-10; Malouin et al., 2007). In all participants, the average rating of the clarity of the image and the intensity of the sensation was at least three (moderately clear image/moderately intense sensation) on a five-point scale.

#### MRI Data Acquisition

Images were acquired at the Cantonal Hospital of Fribourg, Switzerland with a 3 T MRI Scanner (Discovery MR750, GE Healthcare, Waukesha, WI, USA) and a 32-channel standard head coil. The participants were in a supine position throughout the MRI session. The head was stabilized in the coil with cushions in order to minimize head motion.

Functional T2*-weighted images of the whole brain were acquired with Gradient Echo–Echo Planar Imaging sequences (GE-EPI; interleaved axial acquisition from the bottom to the top of the head, voxel size = 1.875 × 1.875 × 3 mm, matrix size = 128 × 128, number of slices = 40, interslice spacing = 0.3 mm, repetition time (TR) = 2500 ms, echo time (TE) = 30 ms, flip angle = 85°, parallel imaging acceleration factor = 2). Blood oxygenation level dependent (BOLD) contrast was used as an index of local increases in brain activity (Kwong et al., 1992). The first 7.5 s of each sequence were discarded to ensure steady-state tissue magnetization. Thus, 150 dynamic volumes were recorded in each scan run.

Additionally, high resolution T1-weighted anatomical scans (FSPGR BRAVO sequence, voxel size = 0.86 × 0.86 × 1 mm, matrix size = 256 × 256, number of coronal slices = 280, no interslice spacing, TR = 7300 ms, TE = 2.8 ms, flip angle = 9°, parallel imaging acceleration factor = 1.5, intensity correction SCIC) were collected for anatomical co-registration.

### Data Processing and Statistical Analysis

#### Balance Performance

Since some participants performed without errors, the data could not be transformed to a normal distribution. Therefore, pre-post differences ([number of errors at post-measurement] − [number of errors at pre-measurement]) were calculated for each participant and groups were compared with Mann-Whitney tests. *Post hoc* comparisons within the groups (pre vs. post) were performed with Wilcoxon tests. The alpha level was set at 0.05. Analyses were performed using SPSS Statistics 24 (IBM Corporation, Armonk, NY, USA).

#### fMRI Data

Processing and statistical analysis of the fMRI data were done with the Statistical Parametric Mapping software (SPM12b, Wellcome Trust Centre for Neuroimaging, University College London, London, UK) running on MATLAB 2015a (The MathWorks Inc., Natick, MA, USA). For better registration, the origin was manually set on the anterior commissure in each image. Standard pre-processing procedures for longitudinal designs implemented in SPM (Friston et al., 2007) were then applied to the functional volumes, including unwarping and realignment, co-registration of the mean anatomical scan of the two sessions to the space of the mean of the realigned functional images, normalization of the images to the Montreal Neurological Institute (MNI) space (2 × 2 × 2 mm voxel size), and finally smoothing with an isotropic 6 mm FWHM Gaussian kernel in order to reduce noise and to correct for between-participants localization differences.

The preprocessed volumes were then subjected to first-level analyses using the general linear model applied on each voxel (Friston et al., 1994; Worsley and Friston, 1995). For each condition (AO+MI and MI), the stimuli were modeled in a block design and convolved with the hemodynamic response function. A high-pass filter (1/128 Hz) and an autoregressive AR(1) model were applied to the time series of each voxel to correct for signal drifts and serial correlations between neighboring voxels in the whole brain, respectively.

For all further analyses, we first calculated subject-based contrasts comparing activity levels during the dynamic balance and the standing task that served as the control task (task contrast) for each session (pre and post). In a next step, we looked at the interaction of session (pre vs. post training) and group (training vs. control group). To do so, interaction contrasts (task contrast * pre vs. post) were calculated on the subject level, separately for the AO+MI and the MI condition. These contrasts were then submitted to random effect analyses in the form of two-sample *t*-contrasts comparing the two groups. The interaction analysis was then followed up by calculating pre-post contrasts for the two groups separately. For this purpose, the individual task contrasts were submitted to second level paired *t*-contrasts (pre vs. post).

Significant effects were assessed at the cluster level using cluster defining height and extent thresholds of *p* < 0.001 (uncorrected at the voxel level) and *k* = 100 contiguous voxels at an FWE rate of *p* < 0.05 (corrected at the cluster level), respectively. The anatomical locations of the clusters were determined based on the Talairach Deamon database (Lancaster et al., 1997, 2000) and the aal (Tzourio-Mazoyer et al., 2002) atlases using the WFU PickAtlas Tool (Maldjian et al., 2003, 2004). All coordinates in this manuscript are presented in the MNI space and all images are displayed in neurological convention.

## Results

### Balance Performance

**Figure 2:**
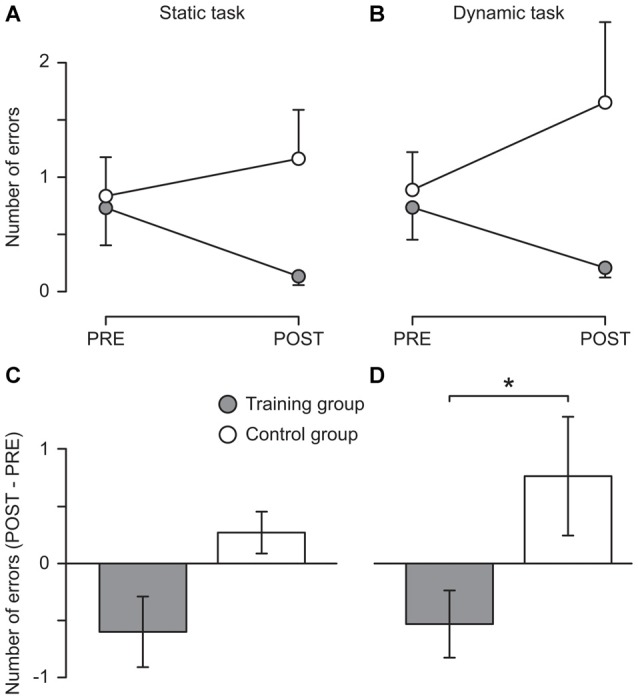
Behavioral results. Mean number of errors committed during 15 s of one-legged stance on stable ground (**A**; Static task) and on a free-swinging platform (**B**; Dynamic task) before (PRE) and after (POST) 5 weeks of balance training (Training group, filled circles) or habitual activity (Control group, open circles). In **(C,D)**, the change from pre to post is displayed for the static and the dynamic task, respectively. An error was defined as touching the ground with the foot of the non-supporting leg or holding on to a handrail. For each participant, the mean of three trials was used. *The change from pre to post differed significantly between groups (*p* = 0.044). Error bars represent the standard error of the mean.

Descriptive results showed that the training group reduced the number of errors in both the static (from 0.7 ± 1.3 to 0.1 ± 0.3) and the dynamic task (from 0.7 ± 1.0 to 0.2 ± 0.3) while in the control group the number of errors slightly increased in both tasks (from 0.8 ± 1.2 to 1.1 ± 1.5 and from 0.9 ± 1.1 to 1.7 ± 2.4, respectively, see Figure 2). These changes were not significant, possibly due to the lack of sensitivity of the clinical tests used. However, Mann–Whitney tests indicated that the difference between the two measurement sessions differed significantly between the two groups for the dynamic task (*U* = 37.50, *z* = 2.01, *p* = 0.044, *r* = 0.41) with a nonsignificant trend also for the static task (see Figure 2). Moreover, the improvements of the training group are likely to be underestimated since many participants (nine and five in the static and the dynamic task, respectively; control group: six and five, respectively) performed without any error during the pre-measurement and thus could not further improve. Furthermore, three participants of the training group could not be included in the analysis of the dynamic task because they were not able to perform the task during the pre-measurement. However, all of them were able to perform the task without any error during the post-measurement.

### Functional Brain Activity

**Figure 3:**
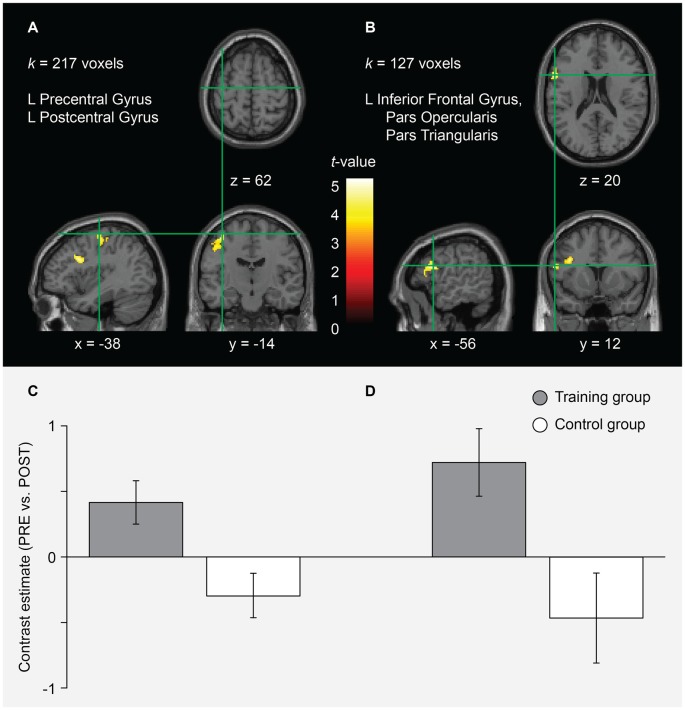
fMRI results for the interaction of session and group in the AO+MI condition. Two clusters **(A,B)** were identified for which the pre-post contrast was significantly greater in the training compared to the control group. For each cluster, the anatomical brain structures over which it extends and the cluster size (*k*) is indicated. Significant effects were assessed at the cluster level using cluster defining height and extent thresholds of *p < 0*.001 (uncorrected at the voxel level) and *k* = 100 contiguous voxels at an FWE rate of *p < 0*.05 (corrected at the cluster level), respectively. The coordinates (numbers and green lines in the images) indicate the position of the local maximum (voxel with the highest *t*-value) within each cluster. Contrasts are presented on a normalized single-subject brain in the montreal neurological institute (MNI) space. The bar graphs **(C,D)** represent the pre-post contrast estimates (and the 90% confidence interval) within each group for the local maximum of the clusters **(A,B)**, respectively. The graphs indicate that these voxels were more activated before the training in the training group while the control group showed an inverse tendency.

In the AO+MI condition, two clusters were found for which the pre-post contrast (greater activity before the training) was significantly greater in the training compared to the control group. In Figure 3 and Table 1, the size, the coordinates of the local maxima and the anatomical brain structures over which it extends are presented for each cluster. No significant group differences were found when looking at the post-pre contrast (greater activity after the training). These findings indicate that balance training led to reductions in brain activity during AO+MI of a balance task in specific areas. According to the atlases used, cluster A covers an area that extends over the left precentral and the postcentral gyri while the area of cluster B can be assigned to the left inferior frontal gyrus (partes opercularis and triangularis), probably extending to the precentral gyrus (see Figure 3 and Table 1). The results of an extensive meta-analysis that used fMRI results of 126 articles to define the locations and boundaries of the motor and premotor cortices (Mayka et al., 2006) were used to identify the voxels that are likely to lie in motor areas. Accordingly, cluster A covers an area that largely lies within M1 and to a lesser extent within the primary somatosensory cortex. Cluster B is situated more anterior, inferior and lateral and can be assigned to the left ventral PMC. Notably, no significant interaction effects were found for the MI condition.

**Table 1:** fMRI results for the interaction of session and group in the AO+MI condition.

Anatomical region	Cluster extent	MNI coordinate			*Z*
Cluster A	217				
L precentral gyrus^a,b^		−38	−14	62	5.28
L precentral gyrus^a,b^		−42	−16	54	4.84
L precentral gyrus^a^		−44	−16	46	4.55
L postcentral gyrus^b^					
Cluster B	127				
L inferior frontal gyrus, pars opercularis^a,b^		−56	12	20	5.01
L inferior frontal gyrus, pars triangularis^a,b^		−54	24	12	4.19
L precentral gyrus^a^		−58	6	14	3.68

*The table displays the clusters for which the pre-post contrast was significantly greater in the training compared to the control group. For each cluster, the coordinates (in the MNI space), the Z-value, as well as the corresponding brain area of up to three local maxima (voxels with the highest *t*-value) that are more than 8 mm apart are indicated. Significant effects were assessed at the cluster level using cluster defining height and extent thresholds of *p* < 0.001 (uncorrected at the voxel level) and *k* = 100 contiguous voxels at an FWE rate of *p* < 0.05 (corrected at the cluster level), respectively. ^a^Based on the Talairach Deamon database atlas. ^b^Based on the aal atlas. L, left; R, right*.

**Figure 4:**
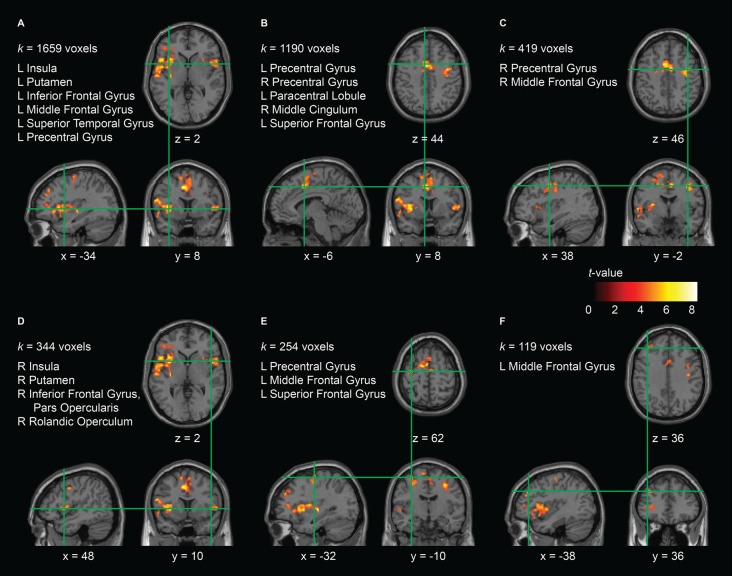
Effect of the balance training on brain activity during AO+MI in the training group. Six clusters **(A–F)** were identified that were significantly more activated before the training than after it. For each cluster, the anatomical brain structures over which it extends and the cluster size (*k*) are indicated. The copdordinates (numbers and green lines in the images) indicate the position of the local maximum (voxel with the highest *t*-value) within each cluster. Significant effects were assessed at the cluster level using cluster defining height and extent thresholds of *p* < 0.001 (uncorrected at the voxel level) and *k* = 100 contiguous voxels at an FWE rate of *p* < 0.05 (corrected at the cluster level), respectively. Contrasts are presented on a normalized single-subject brain in the MNI space.

In order to investigate the interaction effect in the AO+MI condition in more detail, we looked at the pre-post contrast in the training and the control group separately. While for the control group no effects were found, the analysis revealed six clusters that were significantly less activated after the balance training in the training group. The clusters are presented in Figure 4 and Table 2. Cluster A covers a large area (1659 voxels) that extends from the left middle frontal gyrus through the inferior frontal gyrus, the inferior part of the precentral gyrus, and the superior temporal gyrus to the insula and the putamen. Cluster A furthermore covers a considerable part (68 voxels) of cluster B of the interaction analysis (Figure 3) which can be assigned to the ventral PMC. A second large cluster (B; 1190 voxels) mainly covers an area that has been assigned to the left and right SMA. It has further to be noted that cluster E partly covers the area of cluster A of the interaction analysis, namely 27 voxels which can all be attributed to the left M1. According to the article by Mayka et al. (2006), the different clusters cover large regions within primary and secondary motor areas, namely the left and right M1 (cluster 5 and 3, respectively), the left and right pre-SMA and SMA proper (cluster 2), the left dorsal (clusters 2 and 5) and ventral PMC (cluster 1) as well as the right ventral PMC (cluster 4).

**Table 2:** Effect of the balance training on brain activity during AO+MI in the training group.

Anatomical region	Cluster extent	MNI coordinate			*Z*
Cluster A	1659				
L lentiform nucleus^a^		−32	−16	0	8.47
L insula^a,b^		−34	8	2	7.54
L insula^a,b^		−34	18	2	6.94
Cluster B	1190				
L supplementary motor area^b^		−6	8	44	7.71
L supplementary motor area^b^		−2	4	66	7.70
R supplementary motor area^b^		6	0	48	7.22
Cluster C	419				
R precentral gyrus^b^		38	−2	46	7.02
R precentral gyrus^b^		32	−10	48	6.70
R precentral gyrus^b^		46	0	42	5.69
Cluster D	344				
R inferior frontal gyrus, pars opercularis^b^		48	10	2	6.21
R rolandic operculum^b^		54	4	4	5.67
R inferior frontal gyrus, pars opercularis^b^		58	12	4	5.44
Cluster E	254				
L precentral gyrus^a,b^		−32	−10	62	6.96
L middle frontal gyrus^a^		−22	−6	48	5.95
L precentral gyrus^b^		−30	−4	56	5.73
Cluster F	119				
L superior frontal gyrus^a^		−38	36	36	6.56
L middle frontal gyrus^b^					
L superior frontal gyrus^a^		−32	44	32	5.72
L middle frontal gyrus^b^					

*The table displays the clusters that were significantly more activated before the training than after it. For each cluster, the coordinates (in the MNI space), the *Z*-value, as well as the corresponding brain area of up to three local maxima (voxels with the highest *t*-value) that are more than 8 mm apart are indicated. Significant effects were assessed using cluster defining height and extent thresholds of *p* < 0.001 (uncorrected at the voxel level) and *k* = 100 contiguous voxels at an FWE rate of *p* < 0.05 (corrected at the cluster level), respectively. ^a^Based on the Talairach Deamon database atlas. ^b^Based on the aal atlas. L, left; R, right*.

## Discussion

In the present study, we investigated the effect of 5 weeks of balance training on postural stability as well as on brain activity during motor simulation of a challenging balance task in older adults. Descriptive results show that the number of errors was reduced after balance training in the training group, with a significant group difference in the pre-post contrast for the dynamic task. Importantly, this behavioral effect was accompanied by changes in brain activity patterns during motor simulation. Our data showed a significant training-induced reduction in brain activity in areas that are relevant for postural control and, importantly, for which age-related increases in activity, so-called over-activations, have been reported.

Analysis of the interaction of session and group revealed significantly greater deactivations from pre to post in the training group in the left M1 and ventral PMC (see Figure 3 and Table 1). When looking at the training group alone, we found that additional motor areas, such as right M1, left and right pre-SMA and SMA, right ventral PMC, left dorsal PMC, and putamen were significantly less activated after training (see Figure 4 and Table 2). All these areas have previously been shown to be over-activated or disinhibited in older adults when performing/imaging motor tasks (Mattay et al., 2002; Heuninckx et al., 2005, 2008; Coxon et al., 2010; Goble et al., 2010; Allali et al., 2014; Mouthon et al., in press). For instance, an age-related increase in activation in the (pre-) SMA has been found with fMRI during MI of gait (Allali et al., 2014) as well as during physical performance of finger (Mattay et al., 2002) or coordinated hand and foot movements (Heuninckx et al., 2005, 2008; Coxon et al., 2010; Goble et al., 2010). The latter studies further reported increased activity in older compared to young adults in premotor areas as well as in the putamen. Most importantly, in a recent study of our group, comparing pre-measurements of 16 participants of the present study with a group of young adults, we found relative over-activations in older adults in SMA, M1, PMC and putamen during motor simulation of balance tasks (Mouthon et al., in press). Thus, there is evidence that the participants of the training group indeed displayed over-activations before the training. Similarly, TMS studies repeatedly demonstrated increased excitability and reduced inhibition during standing (Baudry et al., 2014, 2015, Papegaaij et al., 2014) or motor simulation of balance tasks (Mouthon et al., 2016) supporting the idea that such age effects can also be found within M1.

The training group further showed reductions in brain activity after the training in clusters covering areas within the left superior temporal gyrus as well as left and right insula. Both superior temporal gyrus and insula have been attributed to the multisensory vestibular cortex (Zwergal et al., 2012). In an fMRI study that looked at differences in brain activity between young and older adults during imagined standing, walking and running (Zwergal et al., 2012), significantly higher activation levels were found in older adults in exactly these areas. Interestingly, these effects were most prominent during imagined standing. The authors hypothesized that these relative over-activations in older adults are the consequence of a reduced reciprocal inhibitory sensory interaction, indicating a more multisensory, conscious postural control strategy (Zwergal et al., 2012). The reduced activity in these multisensory vestibular areas that we found after the training in the present study may indicate a training-related reversion of this aging effect.

Many of the effects were found only in one hemisphere—most of them in the left. This is in line with neurophysiological evidence showing that the left hemisphere is dominant for the execution as well as MI of motor skills, both for unilateral tasks performed with either side and for bilateral coordination tasks (Serrien et al., 2006; Stinear et al., 2006).

In summary, we can say that the physical balance training led to reduced activity during motor simulation of a challenging balance task in brain areas which have been shown to be more activated in older compared to young adults during (simulation of) motor performance. These changes in the brain activity patterns suggest that, in older adults, balance training leads to changes in the internal representation of balance tasks—toward the one observed in young adults. A similar effect has been suggested by Penzer et al. (2015) who found, using electrophysiological techniques such as TMS and peripheral nerve stimulation, adaptations in the neural control of leg muscles during standing after 6 weeks of combined balance and strength training.

We appreciate that physical training certainly influences the ability to mentally simulate motor tasks. In this context, it could be argued that the observed training effects were due to such mechanisms rather than to actual adaptations in the postural control strategy. However, according to Jeannerod’s mental simulation theory (Jeannerod, 2001), this is based on a better internal representation of the motor task—and this can be developed in different ways (MI, AO, AO+MI, or real task execution) by activating the movement-related network. Furthermore, the task that was simulated during the fMRI measurements was not practiced by the training group. All subjects performed the task twice during the measurements (pre and post) and thus both groups had the same experience with the task.

We recognize that from changes in brain activity patterns during motor simulation of a balance task we cannot directly infer such changes during actual execution of the same task. However, as stated in the introduction, there is conclusive evidence that the brain networks activated during motor simulation and actual performance overlap to a substantial extent (Jeannerod, 2001; Eaves et al., 2016). Furthermore, evidence was provided that motor simulation of balance tasks improves postural control during physical balance tests (Taube et al., 2014). Finally, and importantly, the neural adaptations in the balance training group of the present study were accompanied by improvements in postural control. In the context of this close interrelation of mental and physical balance performance, the presented fMRI results confirm and extend previous evidence that balance training in older adults may reverse the well-known over-activation patterns in the neural network (including multisensory vestibular, premotor and motor areas) relevant for the control of upright posture.

Significant changes in brain activity were found only during AO+MI and not during MI alone. This finding is in line with previous studies reporting that the combination of MI and AO is more effective in activating relevant brain areas than either technique alone (Mouthon et al., 2015; Taube et al., 2015; Eaves et al., 2016). The longitudinal data of the present study suggest that AO+MI of a challenging postural task does not only activate relevant brain networks more effectively than MI alone but that brain activity during AO+MI is also more sensitive to training adaptations than during MI alone.

## Author Contributions

JR, AM, MK, MM, J-MA and WT contributed to the conceptualization and the design of the study, critically revised the work for important intellectual content and approved the final manuscript. JR, AM and MK performed all experiments. JR and MM analyzed the data. JR prepared the manuscript.

## Conflict of Interest Statement

The authors declare that the research was conducted in the absence of any commercial or financial relationships that could be construed as a potential conflict of interest.
